# TGF-β Sensitivity Restrains CD8+ T Cell Homeostatic Proliferation by Enforcing Sensitivity to IL-7 and IL-15

**DOI:** 10.1371/journal.pone.0042268

**Published:** 2012-08-06

**Authors:** Lisa D. S. Johnson, Stephen C. Jameson

**Affiliations:** Lab Medicine and Pathology, Center for Immunology, University of Minnesota, Minneapolis, Minnesota, United States of America; Johns Hopkins University School of Medicine, United States of America

## Abstract

The pleiotropic cytokine TGF-β has been implicated in the regulation of numerous aspects of the immune response, including naïve T cell homeostasis. Previous studies found that impairing TGF-β responsiveness (through expression of a dominant-negative TGF-β RII [DNRII] transgene) leads to accumulation of memory phenotype CD8 T cells, and it was proposed that this resulted from enhanced IL-15 sensitivity. Here we show naïve DNRII CD8 T cells exhibit enhanced lymphopenia-driven proliferation and generation of “homeostatic” memory cells. However, this enhanced response occurred in the absence of IL-15 and, unexpectedly, even in the combined absence of IL-7 and IL-15, which were thought essential for CD8 T cell homeostatic expansion. DNRII transgenic CD8 T cells still require access to self Class I MHC for homeostatic proliferation, arguing against generalized dysregulation of homeostatic cues. These findings suggest TGF-β responsiveness is critical for enforcing sensitivity to homeostatic cytokines that limit maintenance and composition of the CD8 T cell pool. (154 words).

## Introduction

Memory phenotype T cells are generated following effective priming of a response to foreign antigens, but can also be induced by other cues, including the response to lymphopenia termed homeostatic proliferation (HP) [1]. Previous studies have indicated that HP of naïve CD8 T cells leads to their acquisition of phenotypic and functional memory CD8 T cells characteristics, including their ability to control pathogen infections much like antigen-primed memory CD8 T cells [2]. While the cytokines IL-7 and IL-15 play a key role in supporting generation and maintenance of both HP and conventional memory CD8 T cells, little is known about factors which negatively regulate memory CD8 T cell homeostasis [3]. Several groups have shown that abrogating transforming growth factor β (TGF-β) reactivity in T cells leads to dramatic T cell expansion, and can lead to autoimmunity and ultimately death [4], [5]. TGF-β can both positively and negatively regulate numerous immune populations, with inhibitory effects on Th1 and Th2 differentiation and positive effects on Treg and Th17 development [6], [7]. In addition, TGF-β has been reported to protect T cells from induction of apoptosis, in certain situations [8], [9], [10]. Complete loss of TGF-β RII or TGF-β1 leads to massive dysregulation in T cell homeostasis and fatal autoimmunity, mediated in part by loss of regulatory T cells [4], [11]. Attenuation of TGF-β signaling has also been achieved by T cell specific expression of a dominant negative (kinase deleted) form of TGF-β RII chain (DNRII) [12], [13]. T cells from such mice exhibit a profound impairment in TGF-β signaling (due to competitive blockade of TGF-β binding) and altered T cell homeostasis, including appearance of memory phenotype CD8 T cells [12], [13]. In one such strain the size of the memory-phenotype CD8 T cell pool is massively increased and these cells are cycling at a higher rate than litter-mate controls [13]. Initial studies suggested these cells were not driven by dysregulated homeostatic proliferation, since breeding to TCR transgenic animals corrected the skewing of the CD8 T cell pool toward memory-phenotype cells [14]. On the other hand, a second study (using a different DNRII transgenic system) suggested that introduction of a TCR transgene delayed but did not completely prevent accumulation of memory phenotype CD44^hi^ CD8 T cells [15]. Previous studies have proposed that DNRII CD8 T cells show enhanced proliferative responses toward IL-15, suggesting TGF-β may typically restrain responses to this homeostatic cytokine [14]. However, no studies have directly assessed the impact of the DNRII receptor on homeostasis of naïve CD8 T cells, or directly tested the requirement for the “homeostatic” cytokines IL-7 and IL-15 in this process.

Hence, the role of TGF-β responsiveness in shaping CD8 T cell responses to homeostatic-driven cues is poorly understood. In this study we analyze OT-I TCR transgenic DNRII T cells and report that impaired TGF-β sensitivity enhances naïve CD8 T cell expansion and production of memory-like cells in response to lymphopenia. Furthermore, homeostatic proliferation of DNRII was also observed in non-lymphopenic hosts and, unexpectedly, was found to be independent of IL-7 and IL-15. Like wild type naïve CD8 T cells, however, HP of DNRII T cells was completely dependent on the presence of self MHC Class I molecules. These data suggest TGF-β sensitivity is important for perception of homeostatic cytokines, acting as a brake on CD8 T cell homeostasis.

## Results

### Impaired TGF-β Reactivity Causes Naïve CD8 T Cells to Ignore Homeostatic Constraints in Lymphopenic and Lymphoreplete Hosts

**Figure 1:**
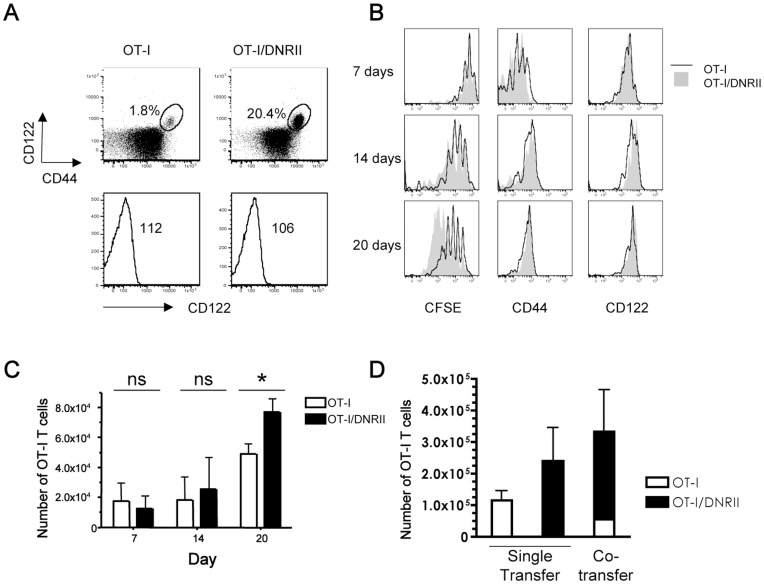
Attenuated TGF-β sensitivity alters homeostasis of OT-I CD8 T cells. (A) Analysis of intact OT-I and OT-I/DNRII mice. Spleen and lymph node cells from OT-I/DNRII mice exhibit an elevated frequency of CD44^hi^CD122^hi^ CD8+ T cells compared to normal OT-I controls (upper panels, gated on CD8+Kb-OVA+). The percentage of CD44^hi^CD122^hi^ is indicated in each dot plot. CD122 express within the CD44^lo^ population is equivalent in OT-I and OT-I/DNRII (lower panels, gated on CD8+ Kb-OVA+CD44^lo^. (B) CFSE labeled CD44^lo^ OT-I or OT-I/DNRII TCD8 cells were adoptively transferred into sublethally irradiated B6 recipients. Host animals were sacrificed at the indicated timepoints and donor cells analyzed. Expression of CFSE, CD44 and CD122 levels on donor OT-I or OT-I/DNRII cells recovered from the spleen at 7, 14, and 20 days after transfer. (C) Mean number of splenic OT-I and OT-I/DNRII cells recovered from the spleen of host animals (n = 3 per group). (D) The mean number of donor cells recovered from single or cotransfer of OT-I and OT-I/DNRII CD8 T cells in the lymph nodes 18 days after transfer into sublethally irradiated B6 (n = 3 per group). All data are representative of at least 3 independent experiments.

A transgene encoding a dominant negative form of the TGF-β RII receptor (“DNRII”) was crossed to the OT-I TCR transgenic background. Polyclonal DNRII mice are characterized by a massive expansion of memory phenotype (CD44^hi^) CD8 T cells [13], but Lucas et al. reported that introduction of the H-Y or 2C TCR transgenes corrected the over-representation of memory-like cells [14], leading to the interpretation that the DNRII memory-like pool arose from immune responses to environmental antigens. In contrast to those findings, we found a high frequency of memory phenotype (CD44^high^/CD122^high^) CD8 T cells in intact OT-I/DNRII mice (Fig. 1a and S1a), although the total numbers of OT-I CD8+ T cells were similar between OT-I and OT-I/DNRII animals (Fig. S1b). The elevated percentage of memory phenotype CD8 T cells in OT-I/DNRII was apparent as early as 3 weeks of age (Fig. S1c), suggesting that DNRII expression leads to dysregulated normal CD8 T cell homeostasis soon after the peripheral T cell pool is established. Adult OT-I/DNRII Rag^−/−^ animals also showed greater frequencies of memory phenotype CD8 T cells, indicating that these results are not a consequence of endogenous TCR rearrangements (Fig. S1d). In order to accurately compare the response of naïve OT-I and OT-I/DNRII cells, all subsequent experiments were conducted using MACS purified CD8^+^CD44^lo^CD122^lo^ T cells (Fig. S1a) in adoptive transfer studies.

In the absence of foreign antigen stimulation, memory-like CD8 T cells can be generated via “homeostatic proliferation” which is induced by physiological and experimental lymphopenia [16]. Lymphopenia induced proliferation is driven by an increased availability of cytokines (notably IL-7) and access to self-pMHC due to eradication of competing host cells [1]. To study the effect of attenuated TGF-β responsiveness on homeostatic proliferation of naïve OT-I T cells, we isolated naïve phenotype (CD44^lo^) OT-I or OT-I/DNRII CD8+ cells and transferred these into sub-lethally irradiated hosts. As expected, naïve OT-I T cells underwent HP, indicated by slow cell division over the first three weeks after transfer, accompanied by upregulation of CD122 and CD44 (Fig. 1b). By around 3 weeks post-transfer, the OT-I/DNRII population showed significantly greater expansion, as detected by increased CFSE dilution (Fig. 1b) and by increased accumulation (Fig. 1c).

**Figure 2:**
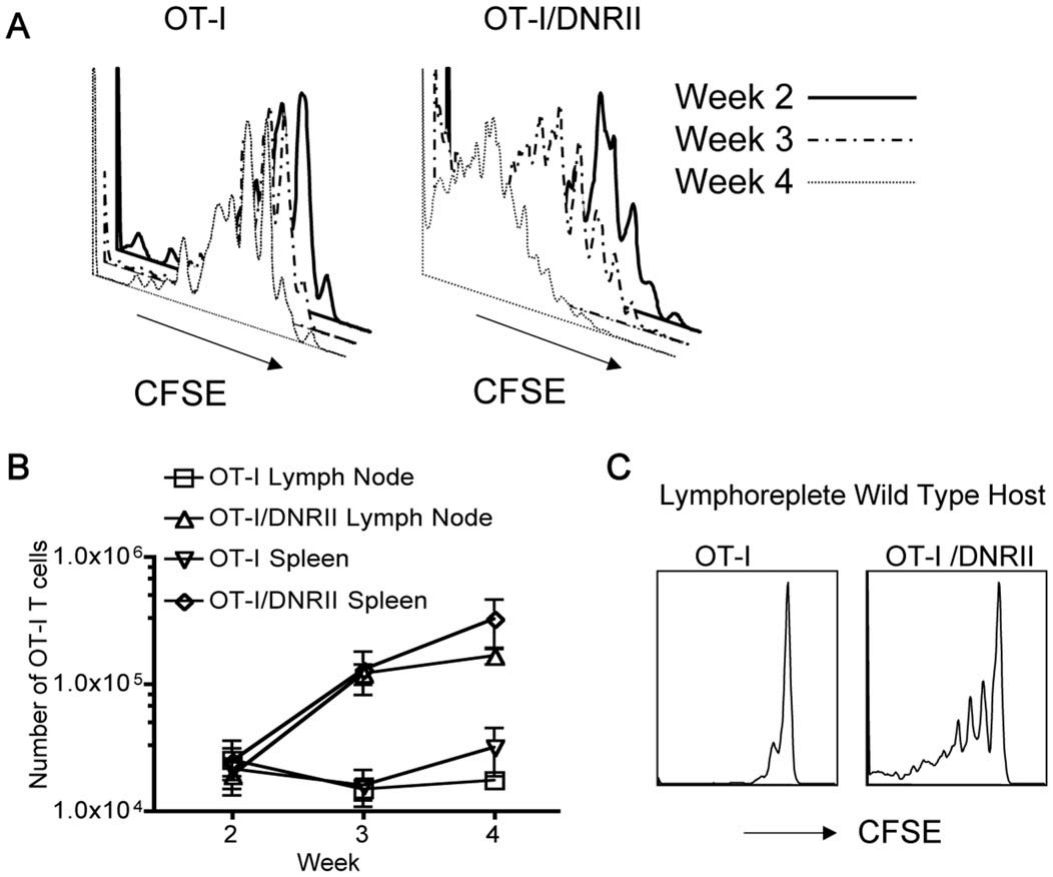
Extended expansion of OT-I/DNRII CD8 T cells as lymphopenic space becomes limiting. CFSE labeled CD44^lo^ OT-I and OT-I/DNRII CD8 T cells were adoptively co-transferred into indicated B6 hosts. Host animals were sacrificed at the indicated timepoints for analysis of donor and host cells. (A, B) OT-I and OT-I/DNRII CD8 T cells were cotransfered into irradiated hosts and analyzed 2, 3 and 4 weeks later. (n = 3 per group) (A) CFSE dye dilution is shown for OT-I cells (left panel), and OT-I/DNRII cells (right panel) CD8 T cells. differentiated by congenic markers. (B) OT-I and OT-I/DNRII CD8 T cell recovery in the lymph node and spleen over time (n = 3 per group). Results are expressed as mean ± SD. (C) CFSE dilution of OT-I and OT-I/DNRII CD8 T cells three weeks after co-transfer into unirradiated (lymphoreplete) B6 hosts. Kb-OVA tetramer pulldowns were used to enrich for OT-I and OT-I/DNRII CD8 T cells. All data are representative of at least 3 independent experiments.

It was possible that the OT-I/DNRII population produced factors that enhanced HP of all T cells in the lymphopenic environment and/or which delayed repopulation of the host lymphoid compartment following sublethal irradiation (allowing for extended homeostatic proliferation of donor T cells). To test whether such non-autonomous effects occurred in our system, we studied the proliferation and expansion of co-transferred OT-I and OT-I/DNRII in lymphopenic hosts. Interestingly, the enhanced proliferation of OT-I/DNRII cells was further exaggerated in the co-transfer setting (Fig. 1d and S2), suggesting a competitive advantage of the DNRII pool over the WT OT-I population, and arguing against an environmental effect induced by the DNRII cells. We also used this approach to investigate the termination of HP in the lymphopenic hosts. Naïve OT-I and OT-I/DNRII cells were analyzed at 2, 3 and 4 weeks after co-transfer into sub-lethally irradiated B6 hosts (Fig. 2a and b). As expected from previous studies [17], OT-I T cell proliferation in irradiated hosts halts between week 2 and 3 post-transfer, as evidenced by lack of substantial additional proliferation or accumulation (Fig. 2a and b). In stark contrast, the OT-I/DNRII pool continues proliferating and accumulating up to week 4 post-transfer (Fig. 2a and b). This continued expansion of OT-I/DNRII T cells was evident despite the reconstitution of the host CD8 T cell pool over the same time course (Fig. S3), indicating the DNRII population has impaired sensitivity to the size of the T cell compartment. Furthermore, the size of the OT-I/DNRII pool was still elevated 3 months after adoptive transfer (Fig. S4), implying that these cells were maintained long after the host T cell pool had been restored. Together, these data suggest that TGF-β signaling sensitivity does not influence initial stages of lymphopenia-induced HP, but has an autonomous role in restraining T cell homeostasis as the T cell compartment fills.

Our finding that naïve DNRII CD8 T cells seemed to ignore normal homeostatic restraints during “refilling” of a lymphopenic environment might suggest that such cells would proliferate even in the absence of lymphopenia. To test this, we adoptively transferred purified naïve CD8 T cells from OT-I or OT-I/DNRII mice into lymphoreplete B6 hosts. As expected, OT-I cells showed minimal proliferation in such hosts after 3 weeks, yet naïve OT-I/DNRII cells underwent numerous rounds of proliferation in this environment (Fig. 2c). Hence, these data support the model that impaired TGF-β responsiveness leads to loss of the normal constraints on homeostatic proliferation induced by a “full” T cell compartment.

### Homeostatic Proliferation of Naïve DNRII CD8 T Cells Occurs in the Absence of the Homeostatic Cytokines IL-7 and IL-15

**Figure 3:**
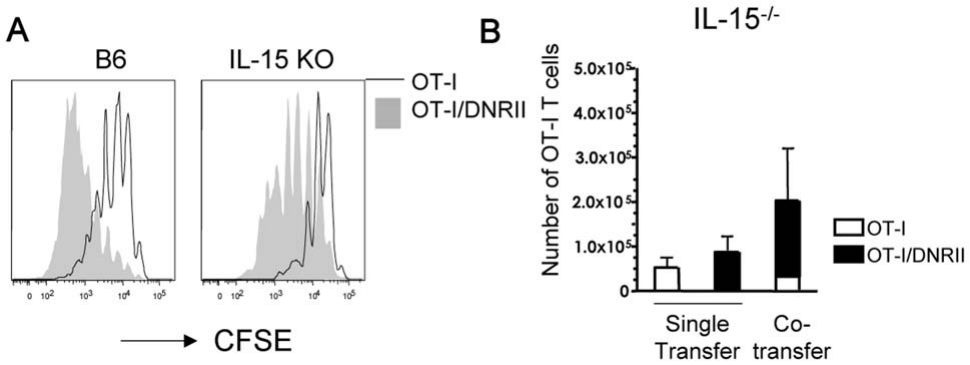
IL-15 is not required for the enhanced homeostatic proliferation of OT-I/DNRII CD8 T cells. CFSE labeled CD44^lo^ OT-I and OT-I/DNRII CD8 T cells were adoptively transferred into sublethally irradiated IL-15^−/−^ hosts. Host animals were sacrificed and donor cells were analyzed. (**A**) CFSE dilution of OT-I and OT-I/DNRII CD8 T cells in the spleen 3 weeks after co-transfer into sub-lethally irradiated B6 and IL-15 KO hosts (n = 3). (**B**) Recovery of OT-I and OT-I/DNRII CD8 T cells 18 days after single or co-transfer in the lymph nodes (n = 3). Data are representative of at least 3 independent experiments.

Lymphopenia-induced proliferation of naïve CD8 T cells is driven by signals through both the TCR (engaging self peptide/MHC ligands) and the cytokines IL-7 and IL-15. Indeed, it was proposed that TGF-β signaling attenuates the memory CD8 T cell response to IL-15 by impairing CD122 expression [14] and, in keeping with this, TGF-β RII-deficient CD4 T cells were shown to exhibit CD122 upregulation suggesting increased IL-15 sensitivity [4]. On the other hand, our studies did not indicate a marked change in CD122 expression in naïve OT-I/DNRII (Fig. 1a) or during HP (Fig. 1b). To explore this model further, we transferred naïve OT-I and OT-I/DNRII CD8 T cells (together or separately) into sub-lethally irradiated IL-15 deficient hosts. As we reported previously, lack of IL-15 in the lymphopenic host leads to reduced expansion of OT-I cells at late stages of the response [17] and this was observed for both WT and DNRII OT-I cells (Fig. 3 and S2). However, OT-I/DNRII T cells still exhibited an advantage compared to their WT counterparts in the IL-15-deficient hosts, as measured by both their proliferation (Fig. 3a) and accumulation (Fig. 3b). As was observed with B6 hosts, OT-I/DNRII had an even greater advantage in situations where OT-I and OT-I/DNRII cells were co-transferred into the same IL-15^−/−^ recipients (Fig. 3b and S2). Together, these data indicate that the competitive advantage of the DNRII pool during HP is not solely due to improved IL-15 sensitivity (although IL-15 enhances the proliferative response of both WT and DNRII populations).

**Figure 4:**
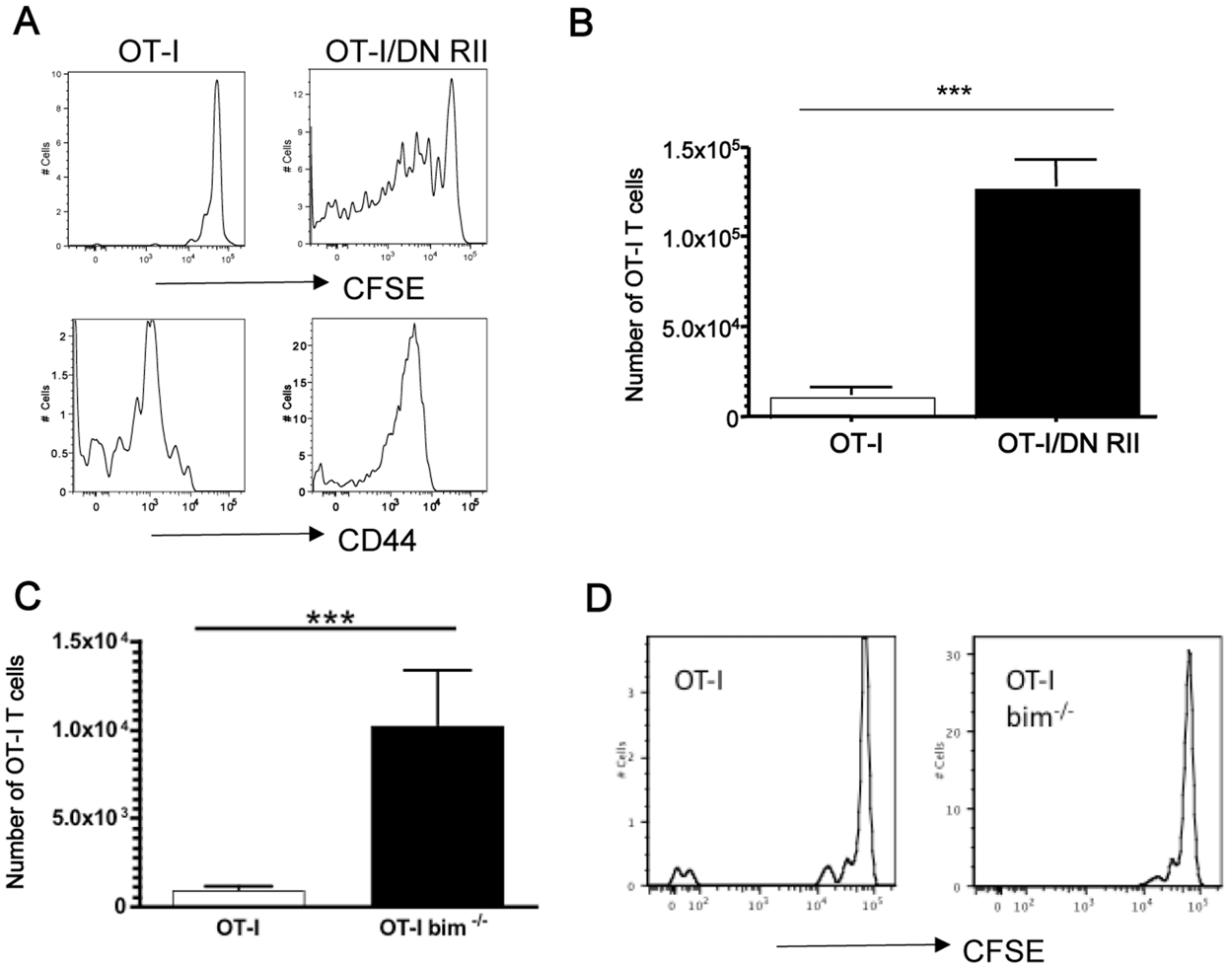
OT-I/DNRII homeostatically proliferate in the absence of both IL-7 and IL-15. (**A,B**) CFSE labeled CD44^lo^ OT-I Rag^−/−^ or OT-I/DNRII Rag^−/−^ CD8 T cells were transferred into sublethally irradiated IL-7^−/−^ x IL-15^−/−^ hosts. After 1 week, spleens were harvested from sacrificed mice and donor cells were analyzed. (**A**) shows representative CFSE dilution and CD44 expression histograms for OT-I and OT-I/DNRII CD8 T cells, as indicated and (**B**) shows recovery of OT-I and OT-I/DNRII 1 week after transfer in the spleen (n = 3 per group). Results in (A,B) are representative of at least 3 independent experiments. (**C,D**) CFSE labeled CD44^lo^ OT-I or OT-I Bim^−/−^ CD8 T cells were transferred into sublethally irradiated IL-7^−/−^ x IL-15^−/−^ hosts and donor cells analyzed in the spleen 1 week later. (C) shows recovery of donor OT-I and Bim^−/−^ OT-I CD8 T cells (n = 5), while (D) shows representative CFSE dilution histograms for these populations. The data in (C,D) are derived from two independent experiments.

These findings lead us to consider the additional role of IL-7, which is the dominant cytokine responsible for naïve T cell homeostatic proliferation in a lymphopenic environment [18]. Mice deficient in both IL-7 and IL-15 do not support homeostatic proliferation of CD8 T cells (whether of naïve or memory phenotype), and competition for these cytokines is a major constraint of T cell proliferation in lymphopenic hosts [1]. As expected then, naïve OT-I T cells transferred into IL-7/IL-15 double knockout (DKO) animals fail to proliferate and remained of naïve phenotype (Fig. 4a), similar to earlier studies [19], [20]. Unexpectedly, however, naïve OT-I/DNRII T cells proliferated extensively in IL-7/IL-15 DKO hosts, and also converted to a CD44^hi^ memory phenotype (Fig. 4a). One week after transfer a significant increase in OT-I/DNRII compared to OT-I was observed indicating that the OT-I/DNRII were also persisting in this environment (Fig. 4b). These data suggest that, rather than enhanced sensitivity to IL-15 (or IL-7), naïve DNRII CD8 T cells are capable of undergoing homeostatic proliferation in the complete absence of these homeostatic cytokines in the host.

Previous studies indicated that TGF-β promotes T cell death by altering the balance of bcl-2 family proteins, including upregulation of the pro-apoptotic factor Bim [10], [21], [22], and the Bim pathway is also involved in T cell apoptosis provoked by cytokine deprivation (including IL-7 withdrawal) [23], [24], [25]. Hence it was possible that the central activity of DNRII was to prevent cell death of CD8 T cells in IL-7/IL-15 DKO hosts, and that proliferation in this environment was driven by other signals. To explore this, we tested the response of OT-I cells lacking Bim. CFSE labeled CD8 T cells from OT-I and Bim-deficient OT-I mice were co-transferred into IL-7/IL-15 DKO hosts, and analyzed for cell recovery and proliferation 7 days later. In comparison to wild type OT-I cells, Bim^−/−^ OT-I CD8 T cells showed enhanced persistence in the IL-7/IL-15 DKO hosts (Fig. 4c), yet did not undergo proliferation in this environment (Fig. 4d). These data indicate that extended maintenance, alone, is not sufficient to allow for proliferation in the IL-7/IL-15 deficient environment, and imply that DNRII expression confers more than a simple survival advantage to CD8+ T cells.

### DNRII CD8 T Cells are Dependent on MHC I for Homeostatic Proliferation

**Figure 5:**
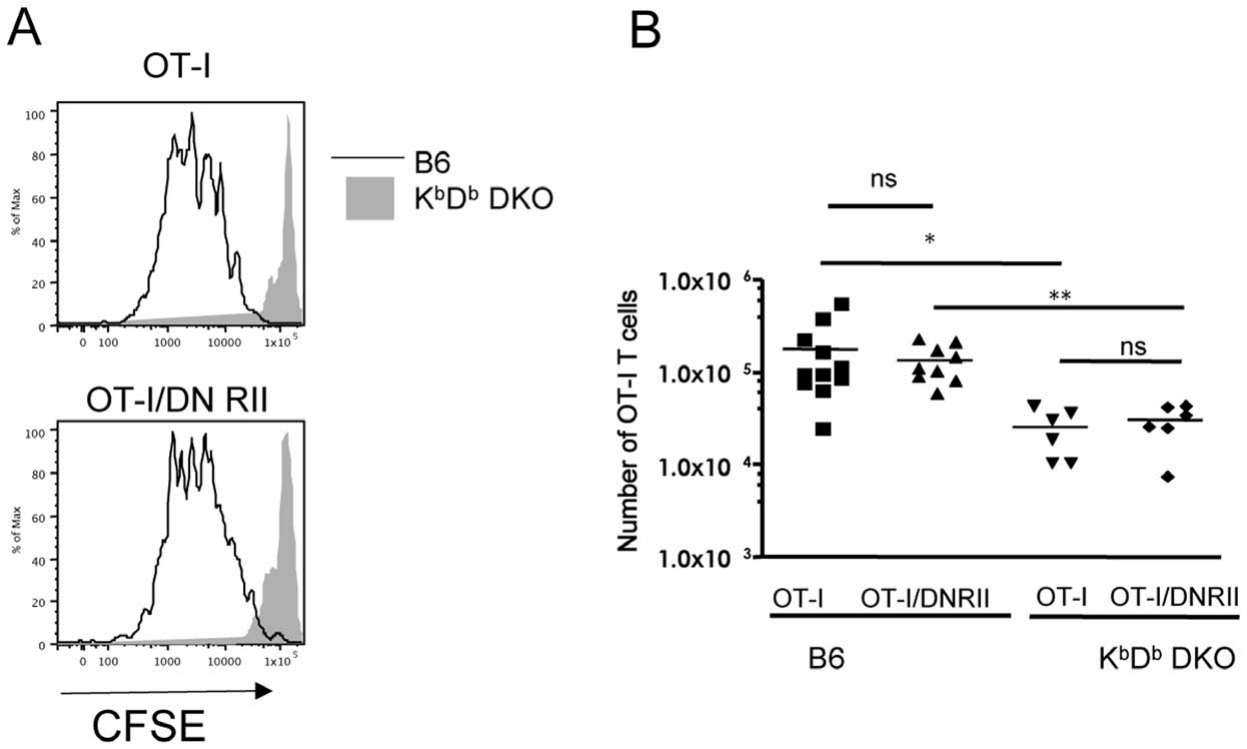
Homeostatic proliferation of OT-I/DNRII is MHC I dependent. CFSE labeled CD44^lo^ OT-I Rag^−/−^ or OT-I/DNRII Rag^−/−^ CD8 T cells were transferred into irradiated K^b^D^b−/−^ or wild-type hosts. One week later, lymph nodes and spleen from sacrificed mice were pooled and analyzed. (**A**) Representative CFSE dilution histograms of OT-I and OT-I/DNRII CD8 T cells in pooled lymph nodes and spleen one week after transfer into irradiated B6 and K^b^D^b−/−^ hosts. (**B**) Recovery of OT-I and OT-I/DNRII CD8 T cells after one week in irradiated B6 or K^b^D^b−/−^ hosts. Each symbol represents an individual mouse. Results are presented as the combination of three independent experiments.

To this point, our data suggested that diminished TGF-β sensitivity released naïve CD8 T cells from the normal cytokine restraints on homeostatic proliferation. However, interaction with self-pMHC molecules is also important in supporting naïve (but not memory) CD8 T cell HP [16]. To test whether DNRII CD8 T cells were independent of self MHC Class I for homeostatic proliferation in vivo, we transferred low numbers of CFSE labeled CD44^lo^ OT-I or OT-I/DNRII CD8 T cells into irradiated K^b^D^b^ deficient mice. As expected, OT-I T cells failed to undergo HP in the Class I MHC-deficient environment showing no CFSE dilution or expansion in stark comparison to their response in irradiated wild-type hosts (Fig. 5). Interestingly, naïve OT-I/DNRII CD8 T cells also failed to expand in Class I MHC deficient hosts (Fig. 5), indicating that altered TGF-β sensitivity does not alter requirement for perception of self MHC molecules in driving homeostatic proliferation. Taken together with the co-transfer studies (Fig. 2 and S2), this suggests that OT-I/DNRII may out compete OT-I for MHC I interaction resulting in the enhanced expansion of OT-I/DNRII. This might suggest enhanced sensitivity of DNRII T cells for TCR stimuli: However, when we assessed naïve OT-I and OT-I/DNRII cells for sensitivity to high and low affinity TCR ligands we found no substantial difference in their short term in vitro reactivity (Fig. S5). Whether DNRII CD8 T cells show increased sensitivity to self peptide/MHC ligands encountered in vivo is difficult to test directly.

Overall, our studies suggest that the competitive advantage demonstrated by naïve DNRII CD8 T cells during homeostatic proliferation is due to their independence from normal homeostatic cytokine restraints, but that the cells retain a requirement for TCR engagement with self MHC ligands. These data then help pinpoint the pathways regulated by TGF-β during normal T cell homeostasis.

## Discussion

Both lymphopenia induced homeostatic proliferation and conventional antigen-induced priming generate functional CD8+ memory T cells, albeit by very different mechanisms [2]. We found that TGF-β reactivity acts as a brake for homeostatic expansion, as evidenced by the sustained proliferation of OT-I/DNRII cells in lymphopenic and non-lymphopenic hosts. Previous studies suggested that the DNRII CD8 T cells showed enhanced CD122 expression and reactivity to IL-15 [14], and the capacity of this cytokine to promote memory CD8 T cell proliferation might suggest an explanation for the increased homeostatic proliferation of OT-I/DNRII. Sanjabi et al. [10] found that during an antigen driven response OT-I/DNRII have an expanded population of short-lived effector cells, a population that is thought to be IL-15 dependent [26]. Altered reactivity to TGF-β might still change IL-15R signaling potential. Indeed, TGF-β signaling in T cells can inhibit c-myc expression [27], and c-myc has been implicated in the proliferative response of CD8 T cells to IL-15 [28]. This model would therefore predict that the competitive advantage displayed by OT-I/DNRII T cells in a lymphopenic environment would be driven by improved IL-15 sensitivity. However, our data indicated that augmented IL-15 sensitivity was unable to account for the proliferative advantage of naïve DNRII cells, since their homeostatic proliferation was also enhanced in IL-15^−/−^ host animals.

Additionally, naïve CD8 T cell homeostatic proliferation is highly dependent on IL-7 (1), and lymphopenic hosts devoid of both IL-7 and IL-15 fail to support homeostatic proliferation of either naïve or memory CD8 T cells [19]. Hence it was surprising that naïve OT-I/DNRII CD8 T cells underwent extensive division in an IL-7/IL-15 DKO environment. Whether DNRII T cells are completely independent of cytokine cues is not clear, and this possibility is difficult to exclude, hence it is possible that additional cytokines (as yet, unidentified) may support HP of DNRII –expressing CD8 T cells. Stromal and myeloid cells are thought to be the critical IL-7 and IL-15 producing populations [29], [30], hence it is unlikely that donor naïve CD8 T cells provide a source for these cytokines. While we cannot completely exclude the possibility that rare cells in the donor cell inoculum produce these factors, our findings clearly show that the requirements for homeostatic proliferation of OT-I/DNRII CD8 T cells differs from that of their wild type counterparts. In contrast, we found that Bim^−/−^ OT-I CD8 T cells resisted apoptosis in IL-7/IL-15 DKO hosts, yet failed to undergo proliferation. From these data, we can conclude that enhanced survival, while it may contribute to the effect of impaired TGF-β sensitivity, is not sufficient to account for the homeostasis of DNRII-expressing CD8 T cells. It is also interesting to note that populations of naïve mouse CD8 T cells distinguished by CD5 expression levels, show distinct capacity for HP and response to homeostatic cytokines [31], [32], although it is not clear whether this phenomenon relates to altered sensitivity to TGF-β signals. Low surface expression of TGF-β RII on CD4+ T cells was implicated in patients with Sézary syndrome [33], suggesting that aberrant expression of surface TGF-β receptor leads to increased T cell proliferation in humans also and it will be interesting to explore whether this type of dysregulated homeostasis involves similar cytokine independence.

We also explored the role of impaired TGF-β sensitivity on TCR signaling requirements during homeostasis. In stark contrast to their loosened cytokine requirements, naïve DNRII CD8 T cells still depended on encounter with self MHC Class I for HP. This raised the possibility that DNRII T cells may exhibit enhanced reactivity toward self pMHC, which might relieve the requirement for homeostatic cytokines. However, our in vitro stimulation assays suggest similar sensitivity of OT-I and OT-I/DNRII naïve CD8 T cells to both high and low affinity TCR ligands (Fig. S5), making this scenario less likely. At the same time, a recent report (published after submission of this manuscript) showed that defective TGF-β reactivity, induced by conditional deletion of TGF-βRII in mature T cells, leads to enhanced autoimmunity in a lymphopenic environment, suggesting that the consequences of self-reactivity can be exacerbated in the absence of TGF-β restraint [34]. In keeping with our findings, those authors showed that OT-I cells with a conditional knockout of TGF-βRII exhibited enhanced HP when transferred into genetically lymphopenic (Rag^−/−^) hosts. Furthermore, Zhang and Bevan confirmed that TGF-βRII deficient naïve CD8 T cells did not display enhanced reactivity to homeostatic cytokines (including IL-7 and IL-15), which we extend in the current report by demonstrating that host derived IL-7 and IL-15 are dispensable for homeostatic proliferation of TGF-β-insensitive naïve CD8 T cells.

Collectively, our data suggest that TGF-β restrains homeostatic proliferation through modulation of responsiveness to both IL-7 and IL-15. Importantly, this effect only becomes manifest late in the response to lymphopenia: At early stages in the response we observed similar expansion of OT-I and OT-I/DNRII CD8 T cells. This can be explained in terms of changing competition for homeostatic cues, which becomes more intense with donor T cell expansion and host T cell reconstitution. In this model, lack of sensitivity to TGF-β allows CD8 T cells to continue expansion as the lymphopenic space is occupied, due to diminished reliance on IL-7 or IL-15 signals. It is unlikely that DNRII-expressing CD8 T cells are oblivious to any cytokine cues, however, and further work to investigate other survival and homeostatic signals is warranted. Indeed, it is important to note that, although the frequency of memory-phenotype CD8 T cells is high in OT-I/DNRII mice, the number of these cells is similar to conventional OT-I animals (Figure S1), suggesting a set-point for TGF-β independent homeostasis.

Our work has potential relevance for the role of TGF-β signals in graft versus host disease (GVHD). TGF-β has been reported to restrain acute GVHD [35], [36], while the response to IL-7 and IL-15 (in the lymphopenic setting induced during bone marrow transplantation) exacerbates GVHD [37], [38], [39]. It will be interesting to test whether enhancing TGF-β signals in T cells would offer an opportunity to limit alloreactive T cell expansion and damage in GVHD.

In summary, our findings indicate that TGF-β acts to enforce naive T cell obedience to homeostatic cytokine cues, hence serving as a restraint on T cell proliferation and differentiation. In this way, TGF-β signals represent a crucial element in preserving diversity and quiescence of the naïve CD8 T cell pool.

## Materials and Methods

### Mice

B6 DNRII transgenic mice [13] were a kind gift of Dr. Ronald Gress (NCI, Bethesda, MD) and were back-crossed to OT-I TCR transgenic mice [40]. OT-I/DNRII were also crossed onto a Rag1^−/−^ background. When OT-I/DNRII Rag1^−/−^ mice were used in experiments, they were compared to OT-I Rag1^−/−^ donor CD8 T cells. OT-I/DNRII and OT-I/DNRII Rag^−/−^ both exhibit increased memory phenotype populations. C57BL/6 (B6) and B6.SJL were from the NCI. C57BL/6 IL-15^−/−^ and H-2K^b−/−^H-2D^b−/−^ (K^b^D^b−/−^) mice were obtained from Taconic Farms (Germantown, NY). IL-7^−/−^ mice were initially provided by Immunex (now Amgen) and IL-7^−/−^ x IL-15^−/−^ animals were kindly provided by Dr. Michael Farrar (University of Minnesota, Minneapolis, MN). These mice were maintained with Uniprim chow (Harlan TEKLAD, Madison WI) fed every other week. Bim^−/−^OT-I mice were kindly provided by Dr. Kris Hogquist (University of Minnesota, Minneapolis, MN). Mice were bred and maintained under specific pathogen-free conditions at the University of Minnesota (Minneapolis, MN). Experiments were conducted with approval by the University of Minnesota Institutional Animal Care and Usage Committee.

### Adoptive Transfer

CD44^lo^ OT-I or OT-I/DNRII were purified by negative selection using MACS columns (Miltenyi Biotec, Bergisch Gladbach, Germany) as previously described [41]. Cells were labeled with carboxyfluorescein diacetate succinimidyl ester (CFSE) as described [42]. We transferred a total of 1×10^6^ CFSE labeled CD44^lo^ OT-I or OT-I/DNRII either as single populations (or as a 1∶1 ratio for double transfer experiments) into B6, IL-15^−/−^ or IL-7^−/−^ x IL-15^−/−^ recipient mice that had been sub-lethally irradiated the day before (420 cGy). In double transfer experiments, the total number of OT-I was kept constant at 1×10^6^ and the total number of OT-I or OT-I/DNRII transferred at 5×10^5^, to avoid increasing the frequency of OT-I cells. For experiments determining MHCI dependency, K^b^D^b−/−^ and B6 mice were given 800 cGy one day prior to adoptive transfer of 5×10^5^ CFSE labeled CD44^lo^ OT-I or OT-I/DNRII. OT-I and OT-I/DNRII were used were either Thy1.2 homozygous or Thy1.1 homozygous or heterozygous, to allow for discrimination between each donor pool and host cells. Lymph nodes and spleens were harvested and pooled for one week after transfer. In experiments where the hosts were not irradiated, transferred cells were enriched using the tetramer pull-down method, as previously described [43]. Depending on the donor/host combinations, donor cells were identified using expression of Thy1.1/Thy1.2, CD45.2 and binding to Ova/K^b^ tetramer.

### Flow Cytometry

Donor and host cells were identified using K^b^-OVA tetramer or by congenic markers along with CD8α (clone 53-6.7; Biolegend). Depending on the experiment, donor cells were identified with CD90.1 (clone HIS51; ebioscience), CD90.2 (clone 30-H12; Biolegend), CD45.1 (clone A2; ebioscience), and CD45.2 (clone 104; ebioscience). The phenotype of donor cells was determined using antibodies for CD44 (clone IM7, BD Biosciences), CD122 (clone TM-β1; BD Biosciences), and CD127 (clone A7R34; ebiosciences). For in vitro stimulation assays, CD69 (clone H1.2F3; ebioscience) was used. Flow cytometry was performed on an LSR II or FACSCalibur (BD Biosciences) and analyzed on FlowJo software (Treestar, Ashland, OR).

### Statistics

When applicable, the data is expressed as the mean ± SD. Unpaired, two-tailed Student’s t- test was performed to determine significance using Prism Version 4.0a software (GraphPad Software, La Jolla, CA). Differences were considered statistically significant when p-values were <0.05. Statistical significance is indicated as follows: *p<0.05, **p<0.01, ***p<0.001.

## Supporting Information

Figure S1 **Characterization of OT-I/DNRII CD8 T cells.** (**a**) The levels of CD44, CD122, CD127, and CD11a were determined for CD8 T cells from OT-I (n = 2), OT-I/DNRII (n = 3) and B6/DNRII (n = 1) 10 week old littermates. MFI are indicated. (**b**) The number of CD8 T cells was determined in the spleen and lymph nodes of mice described in (a) (**c**) CD44 expression on donor OT-I/DN RII CD8 T cells was evaluated prior to MACS purification. N = 20. (**d**) Comparison of CD8 T cells from OT-I Rag^−/−^ and OT-I/DN RII Rag ^−/−^ littermates. The top panel indicates the percentage of Kb-OVA+CD8+ cells within the live gate. The middle panel and bottom panels indicate the percentage of CD44^hi^ and CD122^hi^ cells within the Kb-OVA+CD8+ gate, respectively. Representative of 6 animals per group.
(TIF)

Figure S2 **CFSE dye dilution of OT-I and OT-I/DNRII CD8 T cells in lymphopenic B6 and IL-15 KO hosts.** OT-I and OT-I/DNRII CD8 T cells were transferred either as single populations or as a co-transfer into sub-lethally irradiated B6 (n = 3) and IL-15 KO (n = 3) mice. After 18 days, CFSE dilution was evaluated. Single and co-transfer experiments are each representative of at least 3 experiments.
(TIF)

Figure S3 **Endogenous CD8+ T cell recovery in sub-lethally irradiated wild type mice.** Recovery of CD8 T cells was evaluated 2, 3, and 4 weeks after irradiation and transfer of 1×10^6^ total naive OT-I and OT-I/DNRII. N = 3 for each group.
(TIF)

Figure S4 **OT-I/DN RII CD8 T cells are maintained at an elevated frequency after host has refilled.** OT-I Rag^−/−^ and OT-I/DN RII Rag^−/−^ CD8 T cells were transferred into sub-lethally irradiated hosts and analyzed 3 and 12 weeks after transfer. OT-I/DN RII and OT-I/DN RII Rag^−/−^ CD8 T cells behave equivalently in response to lymphopenia (data not shown). The data are representative of 3 mice per group, from two independent experiments.
(TIF)

Figure S5 **Similar sensitivity of naive OT-I and OT-I/DNRII CD8 T cells to high and low affinity TCR ligands.** CD44^lo^ purified OT-I and OT-I/DN RII CD8 T cells were incubated with the indicated peptide (left panels: SIINFEKL a high affinity ligand for the OT-I TCR; right panels, SIIGFEKL a low affinity ligand for the OT-I TCR) as either individual cultures (top panels) or co-culture (bottom panels). Results are representative of at least 2 independent experiments.
(TIF)
