# Supplementary figures and images for: TGF-β Sensitivity Restrains CD8+ T Cell Homeostatic Proliferation by Enforcing Sensitivity to IL-7 and IL-15
Source: PLoS One. 2012 Aug 6;7(8):e42268. doi: 10.1371/journal.pone.0042268 (PMC3412850; doi:10.1371/journal.pone.0042268)

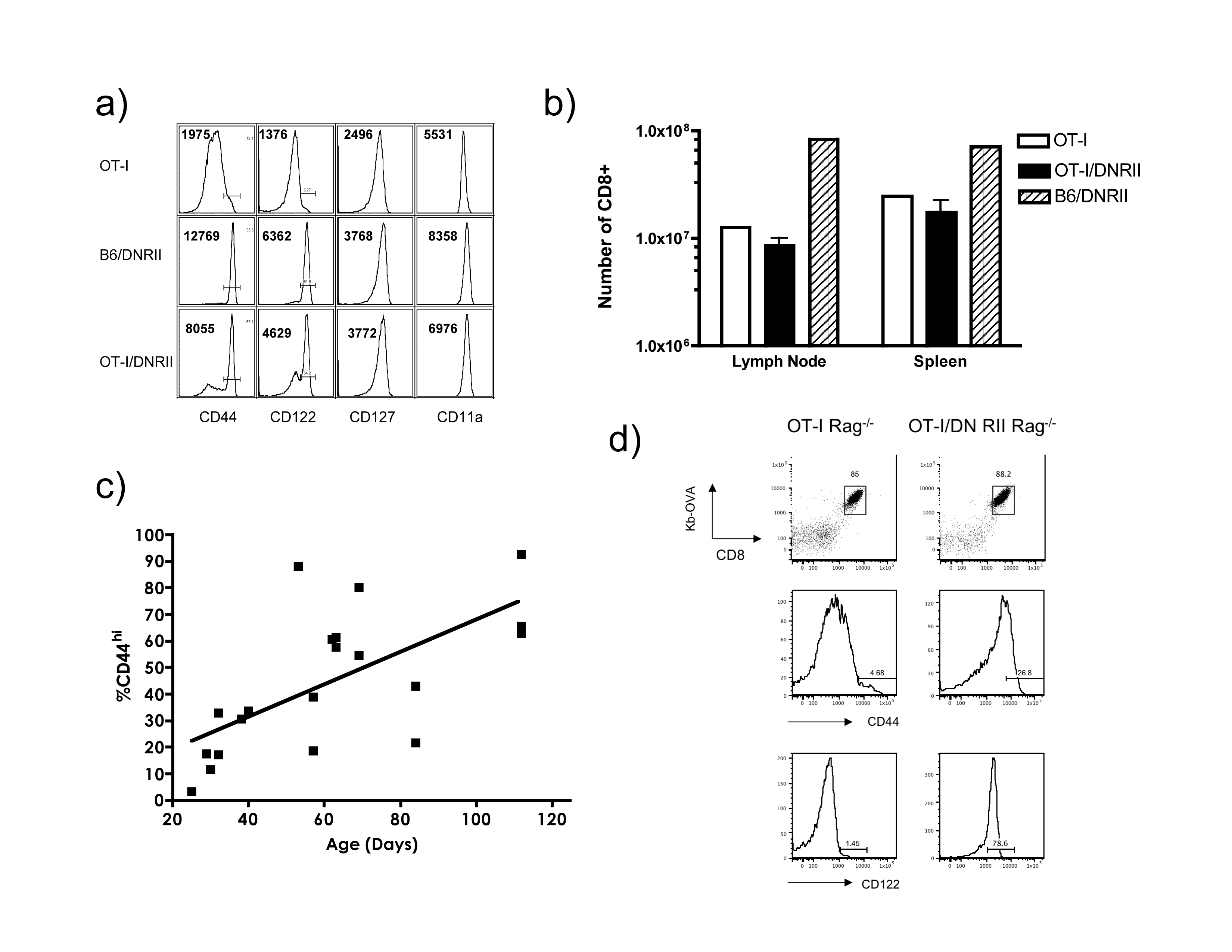

Supplement: Figure S1 — Characterization of OT-I/DNRII CD8 T cells. (a) The levels of CD44, CD122, CD127, and CD11a were determined for CD8 T cells from OT-I (n = 2), OT-I/DNRII (n = 3) and B6/DNRII (n = 1) 10 week old littermates. MFI are indicated. (b) The number of CD8 T cells was determined in the spleen and lymph nodes of mice described in (a) (c) CD44 expression on donor OT-I/DN RII CD8 T cells was evaluated prior to MACS purification. N = 20. (d) Comparison of CD8 T cells from OT-I Rag−/− and OT-I/DN RII Rag −/− littermates. The top panel indicates the percentage of Kb-OVA+CD8+ cells within the live gate. The middle panel and bottom panels indicate the percentage of CD44hi and CD122hi cells within the Kb-OVA+CD8+ gate, respectively. Representative of 6 animals per group. (TIF) [file pone.0042268.s001.tif]

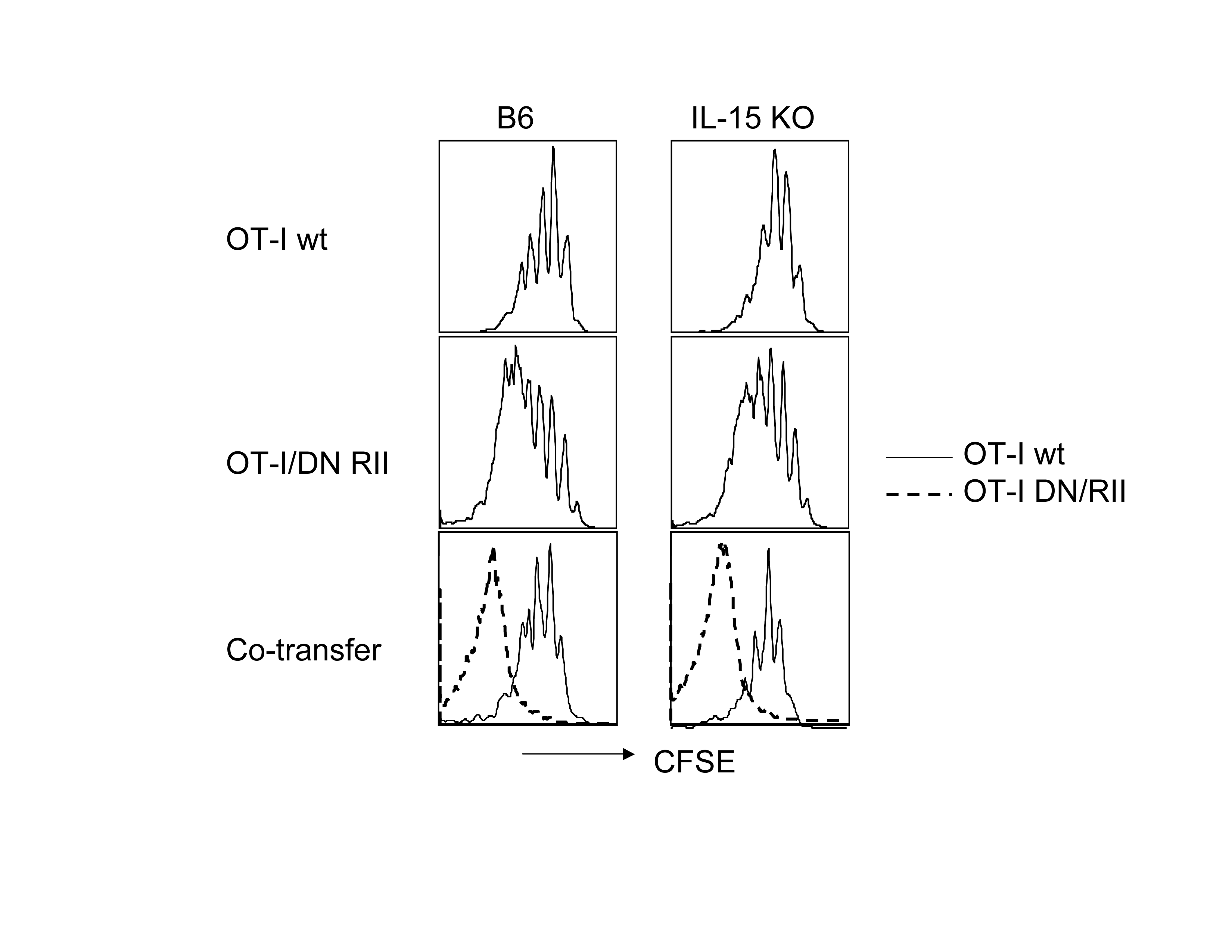

Supplement: Figure S2 — CFSE dye dilution of OT-I and OT-I/DNRII CD8 T cells in lymphopenic B6 and IL-15 KO hosts. OT-I and OT-I/DNRII CD8 T cells were transferred either as single populations or as a co-transfer into sub-lethally irradiated B6 (n = 3) and IL-15 KO (n = 3) mice. After 18 days, CFSE dilution was evaluated. Single and co-transfer experiments are each representative of at least 3 experiments. (TIF) [file pone.0042268.s002.tif]

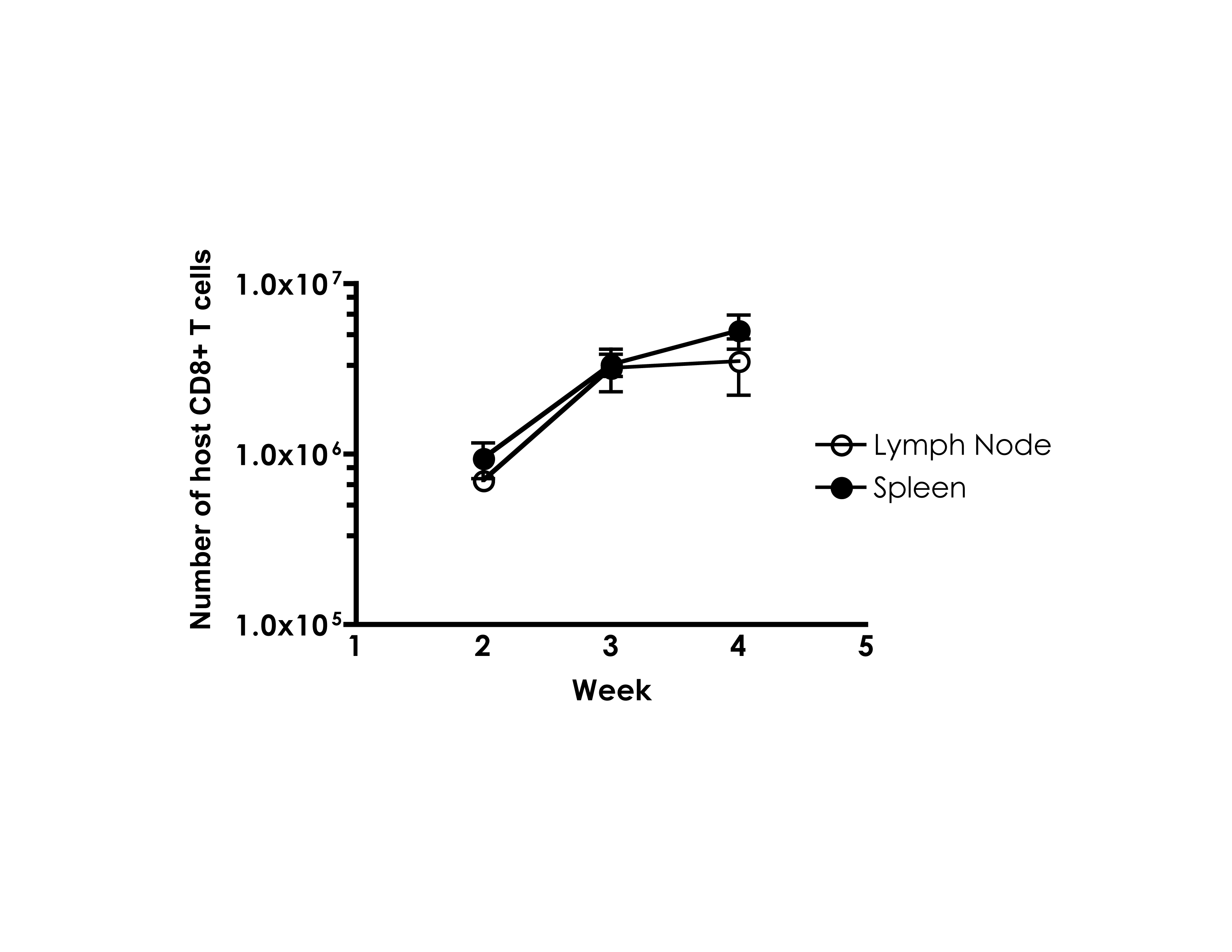

Supplement: Figure S3 — Endogenous CD8+ T cell recovery in sub-lethally irradiated wild type mice. Recovery of CD8 T cells was evaluated 2, 3, and 4 weeks after irradiation and transfer of 1×10^6^ total naive OT-I and OT-I/DNRII. N = 3 for each group. (TIF) [file pone.0042268.s003.tif]

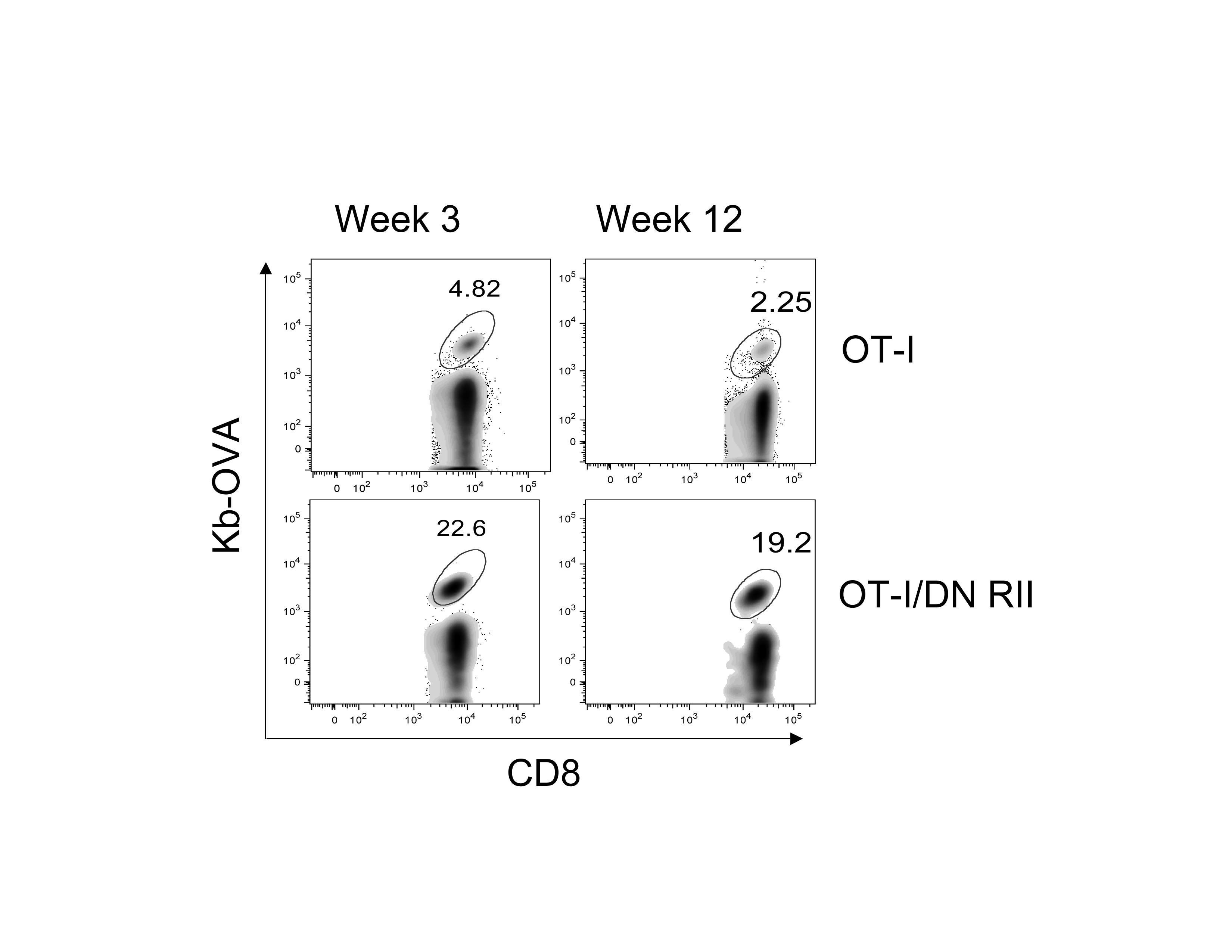

Supplement: Figure S4 — OT-I/DN RII CD8 T cells are maintained at an elevated frequency after host has refilled. OT-I Rag−/− and OT-I/DN RII Rag−/− CD8 T cells were transferred into sub-lethally irradiated hosts and analyzed 3 and 12 weeks after transfer. OT-I/DN RII and OT-I/DN RII Rag−/− CD8 T cells behave equivalently in response to lymphopenia (data not shown). The data are representative of 3 mice per group, from two independent experiments. (TIF) [file pone.0042268.s004.tif]

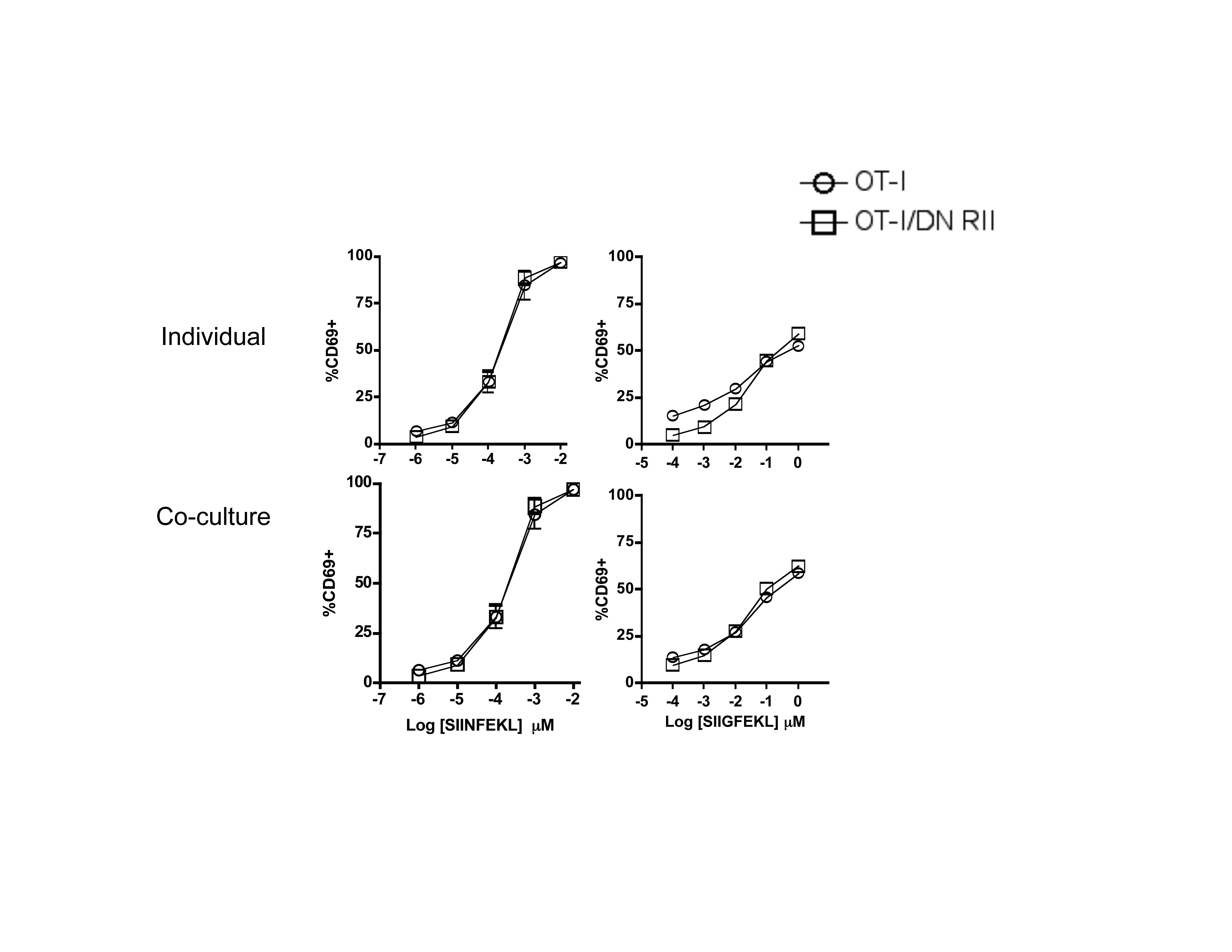

Supplement: Figure S5 — Similar sensitivity of naive OT-I and OT-I/DNRII CD8 T cells to high and low affinity TCR ligands. CD44lo purified OT-I and OT-I/DN RII CD8 T cells were incubated with the indicated peptide (left panels: SIINFEKL a high affinity ligand for the OT-I TCR; right panels, SIIGFEKL a low affinity ligand for the OT-I TCR) as either individual cultures (top panels) or co-culture (bottom panels). Results are representative of at least 2 independent experiments. (TIF) [file pone.0042268.s005.tif]
